# Direct interaction of HIV gp120 with neuronal CXCR4 and CCR5 receptors induces cofilin-actin rod pathology via a cellular prion protein- and NOX-dependent mechanism

**DOI:** 10.1371/journal.pone.0248309

**Published:** 2021-03-11

**Authors:** Lisa K. Smith, Isaac W. Babcock, Laurie S. Minamide, Alisa E. Shaw, James R. Bamburg, Thomas B. Kuhn

**Affiliations:** 1 Department of Chemistry and Biochemistry, University of Alaska Fairbanks, Fairbanks, Alaska, United States of America; 2 Department of Biochemistry and Molecular Biology, Colorado State University, Fort Collins, Colorado, United States of America; George Mason University, UNITED STATES

## Abstract

Nearly 50% of individuals with long-term HIV infection are affected by the onset of progressive HIV-associated neurocognitive disorders (HAND). HIV infiltrates the central nervous system (CNS) early during primary infection where it establishes persistent infection in microglia (resident macrophages) and astrocytes that in turn release inflammatory cytokines, small neurotoxic mediators, and viral proteins. While the molecular mechanisms underlying pathology in HAND remain poorly understood, synaptodendritic damage has emerged as a hallmark of HIV infection of the CNS. Here, we report that the HIV viral envelope glycoprotein gp120 induces the formation of aberrant, rod-shaped cofilin-actin inclusions (rods) in cultured mouse hippocampal neurons via a signaling pathway common to other neurodegenerative stimuli including oligomeric, soluble amyloid-β and proinflammatory cytokines. Previous studies showed that synaptic function is impaired preferentially in the distal proximity of rods within dendrites. Our studies demonstrate gp120 binding to either chemokine co-receptor CCR5 or CXCR4 is capable of inducing rod formation, and signaling through this pathway requires active NADPH oxidase presumably through the formation of superoxide (O^2-^) and the expression of cellular prion protein (PrP^C^). These findings link gp120-mediated oxidative stress to the generation of rods, which may underlie early synaptic dysfunction observed in HAND.

## Introduction

HIV infection of the CNS is characterized by the induction of inflammatory and neurotoxic insults, including the activation of microglia and astrocytes, suspected to stimulate a progressive synaptic degeneration manifested in cognitive decline. Despite the prevalence of HIV-associated neurocognitive disorders (HAND), the underlying molecular and cellular mechanisms promoting pathogenesis remain poorly understood but are thought to consist of a combination of direct viral infection of non-neuronal cells of the central nervous system (CNS) and indirect neurotoxicity mediated by released inflammatory cytokines, metabolites, and viral proteins including the envelope glycoprotein gp120. Gp120 is a potent neurotoxin with roles in a number of indirect and direct neurotoxic pathways. The indirect pathways include the release of excitatory molecules, proinflammatory cytokines, and production of reactive oxygen species (ROS) from activated microglia and astrocytes. Direct effects on neurons arise from NMDAR mediated excitotoxicity and co-receptor mediated neuronal apoptosis from the interaction of gp120 with receptors expressed on the neuronal membrane [1–6].

Gp120 facilitates viral entry to host cells via its interaction with primary host-cell receptor CD4 and chemokine co-receptors CCR5 and CXCR4 at host-cell lipid raft domains [7]. Gp120 co-receptor preference categorizes distinct strains of HIV on the basis of cellular tropism, with macrophage or R5-tropic strains binding CCR5 receptors, T-cell or X4-tropic strains preferentially binding CXCR4 receptors, and dual-tropic strains binding both co-receptors [8]. Coalescence of lipid raft domains into large, stable platforms supports clustering of receptors and components of receptor-activated signaling cascades observed in a number of CNS dysfunctions, including CNS aging and trauma, as well as Alzheimer’s disease (AD) [9–12]. Indeed, gp120 was found to enlarge and stabilize raft domains in a CXCR4-dependent pathway involving the redox-sensitive translocation of neutral sphingomyelinase 2 (nSmase2) to the membrane and the forward trafficking, surface expression, and clustering of NMDA receptors to enlarged raft domains [13–16]. These studies are consistent with macrodomain formation promoted by the release of ceramide from nSmase2-mediated hydrolysis of sphingomyelin to activate signaling in response to various agonists and stress signals. Specifically, a redox-sensitive translocation of nSmase2 is mediated by gp120 stimulating a lipid-raft localized NADPH-oxidase 2 (NOX2) with a subsequent production of superoxide (O_2_^-^) radicals in neurons [13].

The interaction of proteins with lipid-raft localized receptors as a mechanism of regulating pathologic signaling has been observed for a number of neurodegenerative diseases, most notably in AD where soluble, stable amyloid-β dimers and trimers (Aβ_d/t_) interact with the lipid raft-anchored cellular prion protein PrP^C^ to stimulate a pathway mediated by activated NOX leading to the formation of rod-shaped bundles of filaments composed of a 1:1 complex of cofilin-actin [17–19]. These rod-like inclusions are generated in response to oxidative stress conditions and arise from the oxidation of active (dephospho) cofilin in stressed neurons to form intermolecular disulfide cross-linked cofilin [20]. Rods have been described during the progression of AD and other neurodegenerative diseases where they contribute to cytoskeletal abnormalities and synaptic dysfunction through the disruption of normal actin dynamics, the blocking of neuronal transport, and the sequestration of cofilin [21–24].

Given the similarities in the neuronal response to HIV gp120 and that of Aβ_d/t_ it is feasible that gp120-sensitive production of O_2_^-^ mediated by NOX2 is similarly inducing the downstream formation of cofilin-actin rods. Here, we present evidence that gp120 signaling through chemokine co-receptors CCR5 and CXCR4 induces the formation of cofilin-actin rods via a pathway comprised of PrP^C^ and NOX2 common to Aβ_d/t_ and proinflammatory cytokines.

## Methods

### Ethics statement

All animals were handled according to the guidelines provided by the National Research Council for the Care and Use of Laboratory Animals as approved by the Colorado State University Institutional Animal Care and Use Committee (IACUC approved protocol #17-7411A).

### Reagents

All chemical reagents were obtained from Sigma-Aldrich Co. (St. Louis, MO) unless indicated otherwise. Tissue culture reagents and immunocytochemistry reagents were from ThermoFisher (Waltham, MA). Primary antibodies included an affinity purified, rabbit anti-cofilin antibody (1 ng/ml, rabbit 1439) [25], a mouse monoclonal actin antibody (C4, 1:1000, ThermoFisher ICN691002); a rabbit anti-CXCR4 (1:250, NIH Aids Reagent Bank #11232), a rabbit anti-CCR5 (1:250, NIH AIDS Reagent Bank #11236), a rabbit monoclonal antibody (Iba-1, #178846, Abcam, Cambridge, MA) as a microglia marker generously gifted from Dr. R.A. Swanson, Univ. California, San Francisco, a mouse monoclonal antibody 2G13 (Abcam #7762) as growth cone marker, and a mouse monoclonal antibody to glial fibrillary acidic protein (GFAP) (Fisher Sci., MA5-15086). All secondary antibodies (Alexa dye-labeled) were from ThermoFisher (used at 1:500 to 1:1000). HIV1 envelope gp120 glycoproteins, gp120_MN_ and gp120_IIIB_, were obtained from ImmunoDX (Woburn, MA), gp120_CM_ and gp120_LAV_ from ProSpec Proteins (East Brunswick, NJ), and gp120_BAL_ from NIH AIDS Reagent Bank (NIH#4961).

### Neuronal cell culture

Mouse neurons were obtained from the following lines: wildtype C57BL/6, PrP^C^ null (C57BL/6J-Prnp−/−; Talen), and p47^PHOX^ null (B6N.129S2-Ncf1tm1Shl/J p47 phox -/-; JAX 027331). Rat neurons were from Sprague-Dawley rats. Dissociated hippocampal and cortical neuron cultures were prepared either from freshly dissected E16.5 fetal mouse or E18 fetal rat brains according to the method of Bartlett and Banker [26] or from cell stocks of these dissociated neurons slow frozen at 10^6^ cells/ml (hippocampal neurons) or 10^7^ cells/ml (cortical neurons) in 50% fetal bovine serum (FBS), 10% DMSO and self-made Neurobasal medium (see below). Briefly, round glass coverslips (12 mm diameter, #1 German, Carolina Biologicals Supply Co.) inserted into 24 well plates were coated with 100 μg/ml poly-D-lysine in 0.06 M borate buffer (30 min, RT), washed 3 times with ultrapure water, and air dried. Dissociated neurons diluted at least 6 fold in medium immediately after thawing were plated at a density of 40,000 neurons per well (0.5 ml medium per well) in complete growth medium composed of self-made Neurobasal medium supplemented with 10% FBS (Hyclone, VWR Radnor, PA), Glutamax (25 μl/10 ml), 50 U/ml penicillin, 50 μg/ml streptomycin, N21-MAX (1 ml/50 ml, R&D Systems, Minneapolis, MN). Cultures are incubated at 37°C in a humidified 5% CO_2_ atmosphere). After 1 to 2 h, serum-containing medium was removed, replaced with complete growth medium (1 ml/well) and exchanged every 3 days. Self-made Neurobasal was made from all components of commercial NB yet substituted with highly purified L-serine, adjusted final concentrations of 175 μM L-cysteine, 2.5 mM glucose, and an osmolarity of 320 mOsM with NaCl.

### Adenovirus preparation and neuronal infection

The AdEasy system was utilized to generate recombinant, replication-deficient adenovirus (He et al., 1998; Minamide et al., 2003) to express either EGFP-PrP^c^, lacZ-GFP, or a dominant negative mutant of the small membrane NOX subunit p22^PHOX^ (DNp22^PHOX^) in AdTrack vector [19]. Virus titer was determined by immunostaining against E2A in HEK293 cells infected with serially diluted virus as previously described [27]. Titers were expressed as infectious virions/ml (iv/ml) and were usually about 10^9^ iv/ml. Recombinant adenoviruses were stored at -80°C. For optimal infection of primary neurons, recombinant adenovirus was added to dissociated neurons 4 days in vitro (DIV) at a multiplicity-of-infection (MOI) ranging from 30 to 200 to express either EGPF-PrP^c^, lacZ-GFP, DNp22^PHOX^. Infection was executed concomitant with a full medium exchange.

### Rod induction in neuronal cultures

Rod induction was initiated at 6 DIV over a time period of 16 h unless indicated otherwise. After a complete medium exchange (1 ml/well), doses between 250 pM and 750 pM of dual tropic gp120_MN_, X4 monotropic gp120_IIIB_ or gp120_LAV_, or R5 monotropic gp120_CM_ or gp120_Bal_ were added in complete growth medium. Amyloid-β dimer/trimers (Aβ_d/t_) were isolated from medium of 7PA2 cells as previously described [18, 28, 29] and used at ~1 nM final concentration (monomer equivalent) determined from Western blots with monomeric synthetic Aβ standards.

### Pharmacological treatments

Maraviroc (100 nM, Santa Cruz Biotechnology) and AMD3100 (50nM, Santa Cruz Biotechnology) were used as specific antagonists of CCR5 and CXCR4 receptors, respectively. NOX2 was inhibited with TG6-227 kindly provided by Dr. J. D. Lambeth, Emory University [19]. Pharmacological inhibitors were added to cultures 1 h prior to gp120 exposure and maintained for the duration of the experiment.

### Immunolabeling of rods and chemokine receptors

Following treatments, neuronal cultures were fixed in 4% formaldehyde, 0.1% glutaraldehyde in phosphate buffered saline (PBS) (37°C at addition, 30 min, RT). Cultures were washed 3 times with PBS and permeabilized with methanol (chilled to -20°C) for 3 min. Permeabilization with non-ionic detergents must be avoided for best rod preservation. After several washes with Tris buffered saline (TBS), cultures were treated with blocking buffer (5% goat serum, 1% BSA in TBS) for 1 h prior to the addition of primary antibodies (4°C, overnight). After 3 washes with TBS, Alexa-labeled secondary antibodies (1:500 to 1:1000) were applied (1h, RT), followed by 3 washes in TBS. Coverslips were mounted onto glass slides with ProLong Gold Antifade containing DAPI (Fisher Scientific). For immunostainings targeting only surface co-receptors CXCR4 or CCR5, the permeabilization step was omitted.

### Rod quantification

Immunolabeled neurons were imaged on an inverted fluorescence microscope using a 40x 0.95 na air objective and scored by an individual blinded to the treatments or by more than one individual. For most experiments, at least 100 neurons per coverslip were scored for the presence of rods with triplicates for each condition and three independent experiments. Neuronal processes in the vicinity of non-neuronal cells and rod-like staining in growth cones were disregarded in the analysis. Density of neuronal cultures required two ways of quantifying rod formation. Scoring in low-density cultures was performed by calculating the percent of isolated neurons with rods, whereas in higher density cultures, rod index was scored by counting the number of rods per total nuclei (DAPI) per field or number of rods per neuron per field.

### Fluorescence recovery after photobleaching (FRAP)

FRAP experiments were performed on 6 DIV mouse hippocampal neurons infected 60 hrs prior to imaging with adenovirus for expressing cofilin-mRFP and treated with 1 nM Aβ_d/t_ 24 hr prior to imaging. Imaging was performed on an Olympus IX83 microscope with a Yokagawa spinning disc confocal system and a phasor light illumination system (3I, Denver, CO). A 100x, 1.45 na objective was used for photobleaching with either the 488 nm or 561 nm lasers at 100% power for 0.5 s. Single plane images of both emissions were acquired every 5 s using an exposure of 100 ms with laser power at 20%. Acquisition duration was 20 min or until fluorescence recovery plateaued as observed via real-time Slidebook (3I, Denver, CO) fluorescence recovery graphs.

### Statistical analysis

Rod quantification experiments were performed in triplicate for each condition and repeated in three independent experiments. For rod analysis, both rod index (rods per DAPI positive nuclei in a field of view) and/or percent neurons with rods were calculated. Independent group averages obtained from triplicates were used to calculate standard deviations shown on plots. Significant differences among treatments and between treatments and controls were tested using by one-way ANOVA with Tukey’s or Dunnett’s posthoc-analysis using Graph Pad Prism software (GraphPad Software, Inc.). An alpha level of 0.05 was used for statistical significance unless indicated otherwise.

## Results

### Gp120 interacts with neurons to induce actin-cofilin rods in a dose- and time-dependent manner

Prior to experiments examining gp120-mediated rod induction, we first addressed the issue of spontaneous rod formation in neuronal cultures. Spontaneous rods in neuronal processes are commonly observed in fewer than 5% of neurons (rod index < 0.2 rods/neuron) in dissociated hippocampal or cortical cultures under control conditions likely due to culture stress arising from commercial neurobasal medium (e.g. [19]). Since over time, levels of spontaneous rod formation became increasingly variable and reached levels has high as 25% (rod index > 0.5), we sought to improve culture conditions to minimize spontaneous rod formation. Modifications to various media components achieved a reset of spontaneous rod formation to < 5% (rod index < 0.2) using a self-made neurobasal medium composed of L-serine with minimal D-serine contamination, a glucose concentration of 2.5 mM, and a physiological osmolarity of 320 mOsM (S1 Fig). All experiments were executed using self-made neurobasal with the exception of those examining the role of NOX activity.

**Fig 1:**
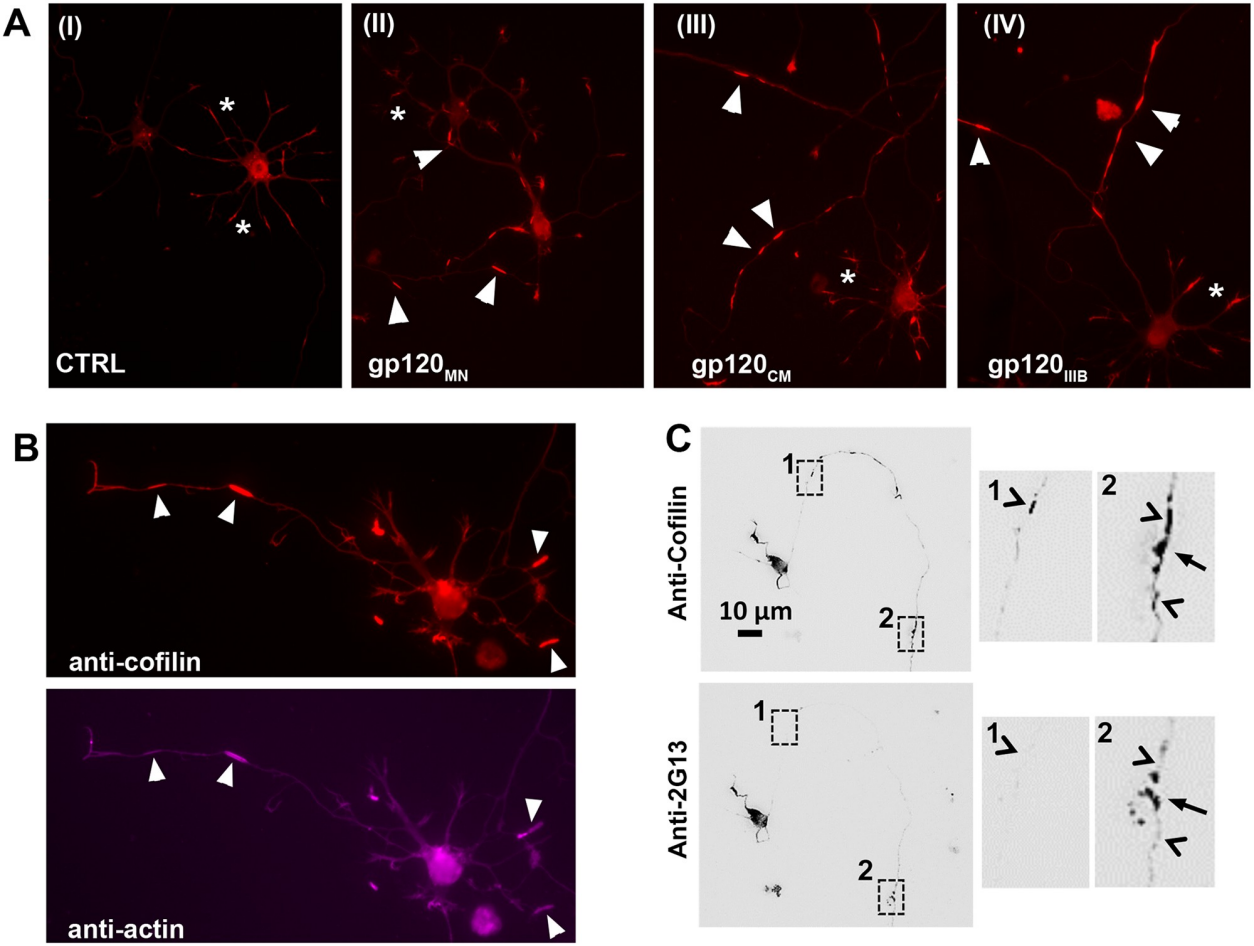
HIV gp120 induces the formation of aberrant, rod-shaped cofilin-actin inclusions (rods). After 18 h of gp120 exposure, dissociated mouse hippocampal neurons cultures were immunostained for cofilin, actin, and/or the growth cone antigen 2G13. (A) Dual tropic gp120MN (II), R5-tropic gp120 CM (III), or X4-tropic gp120IIIB (IV) at 250 pM caused a robust induction of rod-shaped cofilin-actin inclusions (arrow heads) in neurites neurons compared to untreated control (I). Cofilin immunoreactivity in growth cones is indicated by asterisk. (B) Rod-shaped inclusions were immunostained for both cofilin and actin (arrowheads). (C) Notably, the growth cone antigen 2G13 reveals growth cone-associated cofilin indicated by arrows in expanded boxed areas 1 and 2, structures clearly distinct from rods (arrowheads), which are in regions not immunoreactive to the growth cone antigen.

Gp120 was demonstrated previously to be a potent stimulator of ROS production [13]. Given the requirement of oxidized cofilin for the formation of rods (1:1 complex of cofilin:actin), we examined whether dual-tropic gp120_MN_, R5 tropic gp120_CM_, and X4 tropic gp120_IIIB_ were capable of inducing rod formation in cultured mouse hippocampal neurons (Fig 1). After exposure of DIV 6 neuronal cultures with 250 pM gp120 for 18 h, cultures were fixed, immunostained for cofilin, actin, and/or the growth cone marker 2G13, and evaluated for rod formation. Robust rod formation in neuronal processes was evident following treatment with dual tropic or monotropic gp120 (Fig 1A). Since rods are composed of 1:1 oxidized cofilin:actin complexes [17, 20], we demonstrated co-immunostaining against both cofilin and actin (Fig 1B). To demonstrate the different dynamics between cofilin-actin bundles in rods and in growth cones, we performed FRAP analysis (S2 Fig). Recovery of cofilin fluorescence to 50% in growth cone bundles was on the order of 1 min whereas recovery in rods needed to be extrapolated from data to estimate 50% recovery in 80 min confirming the need to remove growth cone cofilin-actin co-stained bundles from rod analysis. To distinguish rods from cofilin-stained actin bundles in growth cones, cultures were double immunostained for cofilin and the growth cone marker 2G13 (Fig 1C). Whereas growth cones are immunoreactive for both cofilin and 2G13, rods are quantified exclusively from regions not immunoreactive for 2G13.

Rod formation was not an exclusive response of mouse hippocampal neurons to gp120. In fact, hippocampal neurons as well as cortical neurons from both mouse and rat exhibited similar rod formation to dual and mono-tropic gp120 as well as Aβ d/t or TNFα (Table 1).

**Table 1:** Rod induction in rodent CNS neurons in response to extrinsic stressors.

Species	Neuronal cell type	Stress	Concentration	% Neurons with Rods		
				Mean	St.Dev.	p>0.05
mouse	hippocampal	---	---	7.30	5.20	
mouse	hippocampal	gp120_MN_	500 pM	28.52	7.46	*
mouse	hippocampal	Aβ d/t	200 pM	24.1	4.7	*
mouse	hippocampal	TNFα	50 ng/ml	25.1	5.8	*
mouse	cortical	---	---	10.81	2.89	
mouse	cortical	gp120_MN_	500 pM	25.96	10.08	*
mouse	cortical	Aβ d/t	1 nM	24.8	5.85	*
rat	hippocampal	---	---	0.33^#^	0.577	
rat	hippocampal	gp120_MN_	500 pM	29.65	8.36	*
rat	hippocampal	Aβ d/t	1 nM	21.67	2.08	*
rat	cortical	---	---	0.25^#^	0.16	
rat	cortical	gp120_LAV_	250 pM	1.27^#^	0.10	*
rat	cortical	gp120_CM_	250 pM	0.53^#^	0.05	*
rat	cortical	gp120_MN_	500 pM	22.82	5.64	*
rat	cortical	Aβ d/t	1 nM	15.36	3.19	*
rat	cortical	TNFα	50 ng/ml	23.06	5.99	*

gp120_MN_ = dual tropic; gp120_LAV_ = X4 tropic; gp120_CM_ = R5 tropic.

Dissociated rodent neuron cultures were exposed on DIV 6 to extrinsic stressors (gp120, Aβ_d/t_, TNFα) at concentrations provided for 18 h prior to fixation and immunostaining against cofilin to visualize rods. Rod induction was quantified as % neurons with rods unless indicated otherwise by # referring to rod index (*p<0.05).

**Fig 2:**
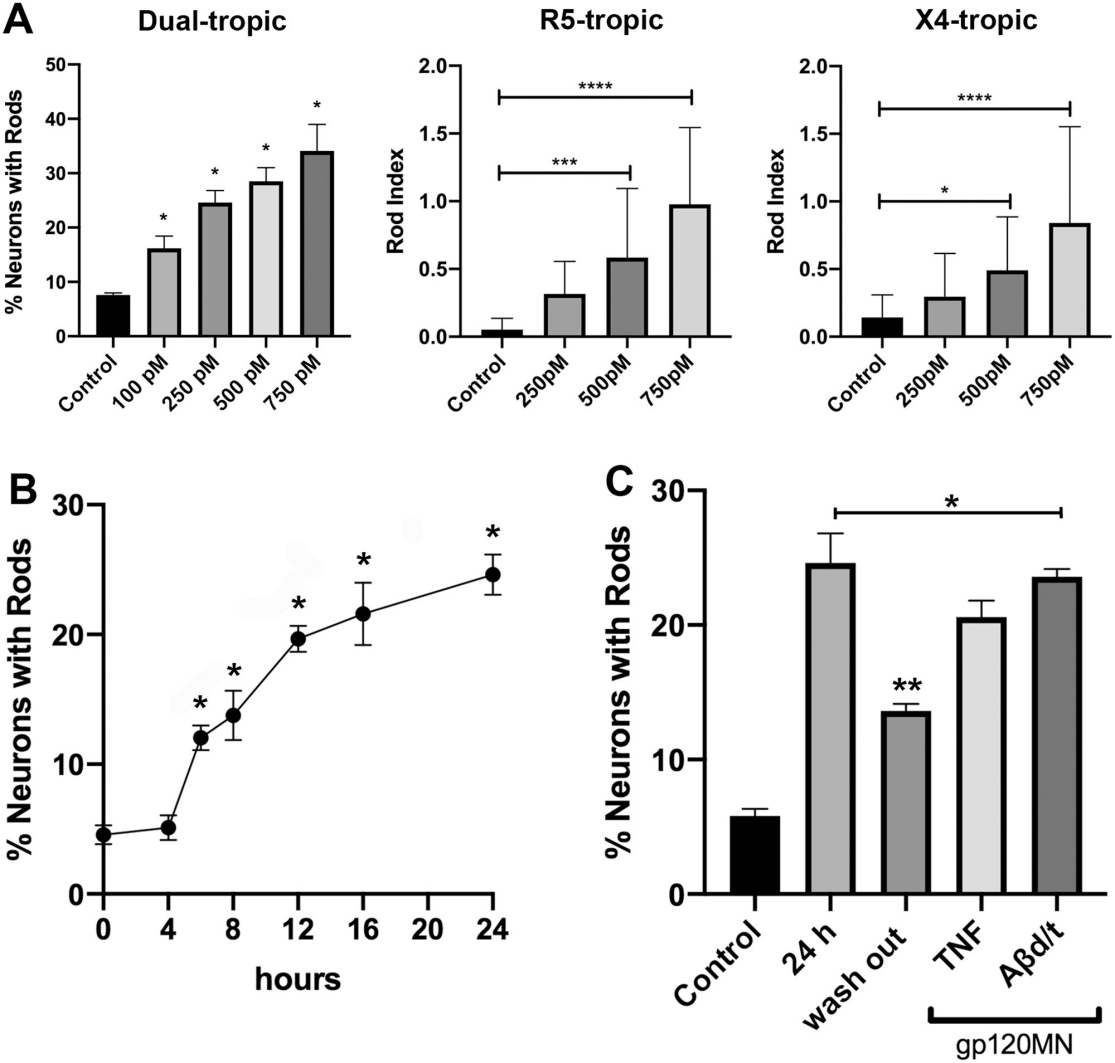
Different tropic gp120 strains induce dose- and time-dependent rod formation in hippocampal neurons. (A) Increasing concentrations of dual tropic gp120_MN_, R5-tropic gp120_BaL_, or X4-tropic gp120_IIIB_ revealed a dose-dependent formation of rods in processes of hippocampal neurons quantified either as the percentage of neurons forming rods (dual-tropic) or as rod index (* p<0.01, *** p = 0.0002, **** p<0.0001). (B) Time-course of rod formation measured as a percent of neurons with rods upon exposure to 500 pM dual-tropic gp120_MN_. Rod induction was significantly above control from as early as 6 h and remained sustained for the duration of the experiment (*p<0.01). (C) A wash-out of gp120_MN_ after 20 h for a 4 h time period significantly reduced rod formation (**p<0.01 compared to no wash-out for 24 h). It is noteworthy that no additive effect was measured upon incubation of hippocampal neurons with a combination of 500 pM gp120_MN_ and 50 ng/ml TNFα or 500 pM gp120_MN_ and 1 nM Aβ_d/t_. Note, all manipulations showed significant rod formation compared to control (*p<0.01).

Rod formation in response to gp120 exhibited both dose and time-dependence (Fig 2). Dual tropic gp120_MN_ induced rod formation significantly above the untreated control for each concentration tested ranging from 100 pM to 750 pM (Fig 2A). As dual-tropic gp120_MN_ is capable of binding to both CCR5 and CXCR4 receptors, we further evaluated the ability of mono-tropic gp120 strains to induce rod formation. Neurons exposed to increasing concentrations of R5-tropic gp120_BaL_ or X4-tropic gp120_IIIB_ for 18 h exhibited rod formation significantly above control at concentrations of 500 and 750 pM for both strains tested, suggesting that both co-receptors are capable of initiating gp120-mediated rod formation. We did not determine a maximum saturation concentration for either gp120 strain since concentrations above 750 pM exhibited significant cytotoxicity over the 18 h incubation period. A time-dependent exposure of mouse hippocampal neurons to dual tropic gp120_MN_ (500 pM) demonstrated significant rod formation in neurites above control by 6 h with a half-maximum rod induction at 8 to 9 h (Fig 2B). A similar time-dependence of rod formation induced by R5-tropic gp120_CM_ and X4-tropic gp120_LAV_ was found in rat cortical neurons. Previous studies have demonstrated that almost all neurons can form rods when subjected to energy depletion [22] but rods are induced in a maximum of 20–25% of cultured mouse hippocampal neurons by Aβ_d/t_ and the proinflammatory cytokine TNFα when used separately or in combination, suggesting that both stressors elicit rod formation through the same pathway [19]. To determine if dual-tropic gp120 is also inducing rods through this same pathway, mouse hippocampal neurons were treated with 500 pM dual tropic gp120_MN_ alone or in conjunction with either 50 ng/ml TNFα or 1 nM Aβ_d/t_ for 24 h before immunostaining and rod quantification. Rods were observed in 20–25% of gp120-treated neurons alone or in combination with Aβ_d/t_ or TNFα (Fig 2C). Interestingly, we also found gp120-mediated rod formation to be reversible since washing out gp120 after a 20 h exposure significantly reduced the percentage of neurons with rods detected 4-hours post washout similar to what was observed for TNFα-induced rods [19] and Aβ_d/t_-induced rods [30].

**Fig 3:**
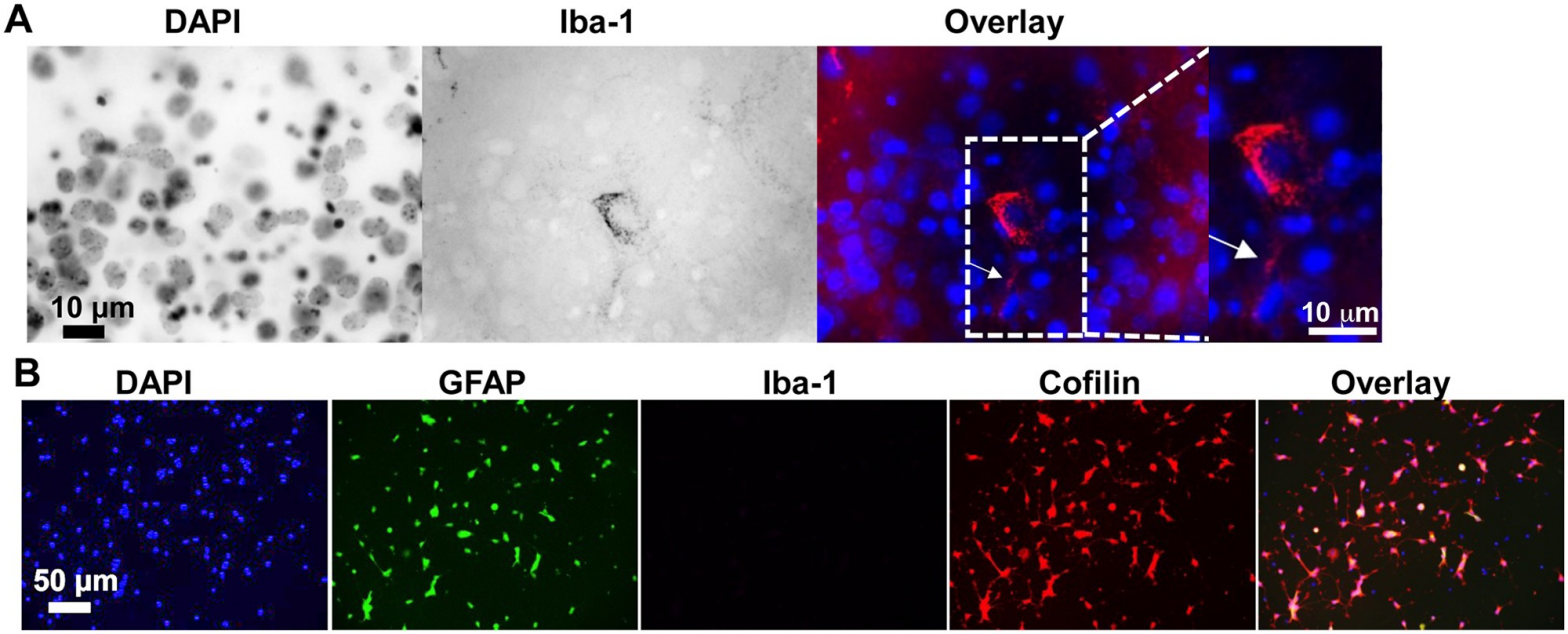
Microglia cells are virtually absent in dissociated cultures of hippocampal neurons. Microglia and astrocytes in dissociated cultures of mouse hippocampal neurons were characterized by immunoreactivity against Iba-1 or GFAP, respectively. (A) Immunostaining of adult mouse brain slices with Iba-1 antibody revealed the presence of microglia indicating that our fixation, methanol-permeabilization and immunostaining protocol worked well for the Iba-1 antibody. Immunoreactivity is shown in the cytoplasm of a microglial cell co-stained with DAPI (nucleus). A ramified extension of the microglial cell is visible (arrowhead in overlaid image, 100x confocal microscopy). Identical staining was obtained in slices following the more extensive but unnecessary citrate buffer antigen retrieval [35]. (B) Dissociated cultures of mouse hippocampal neurons (DIV 7) were fixed, methanol permeabilized and immunostained (identical conditions as in panel A). Confocal images were acquired (20x) to reveal nuclei (DAPI), astrocytes (GFAP), microglia (Iba-1), and cofilin and an overlay image generated. No Iba-1 staining was observed indicating cultures were devoid of microglia, a finding confirmed by scanning with 60x and 100x objectives as well. GFAP-positive cells (astrocytes) comprised about 40% of total DAPI nuclei within this particular preparation.

Productive infection by HIV in the CNS is restricted primarily to microglia and, to lesser extent astrocytes with subsequent injury and apoptotic death in neurons [2, 31, 32]. Hence, there has been some debate whether gp120-associated neuronal injury observed in HAND is the result of indirect effects mediated by the release of neurotoxic, proinflammatory host factors from infected or activated glial cells, or rather the result of a direct neurotoxic effect of soluble HIV proteins shed from infected host cells and virus [12, 33, 34]. For that reason, we examined the presence of microglia cells and/or astrocytes in our neuronal cultures by immunocytochemistry (Fig 3). Anti-Iba1 immunostaining, specific for microglia was first confirmed in adult mouse brain slice cultures revealing cells with ramified extensions characteristic for microglia (Fig 3A). Notably, immunoreactivity against Iba-1 in dissociated mouse hippocampal neuron cultures was absent strongly suggesting cultures were devoid of microglia cells (Fig 3B). In contrast, astrocytes revealed by GFPA immunoreactivity comprised roughly 40% of total cells.

Taken together, these findings suggest that dual tropic and mono-tropic gp120 elicit rod formation in a subpopulation of hippocampal neurons through direct interactions with chemokine receptors with no indirect neurotoxic contribution from microglia. We previously demonstrated no increase in proinflammatory cytokine production in similar cultures [19], suggesting astrocytes are not participating via that route. Moreover, the fact that gp120, regardless of co-receptor tropism, as well as Aβ_d/t_, and TNFα all induced rod formation in the identical population of neurons implies a likely common pathway, and a requirement of continuous exposure to gp120 for the persistence of gp120-induced rods.

### Gp120-induced rod formation is mediated by CCR5 and CXCR4 chemokine receptors

**Fig 4:**
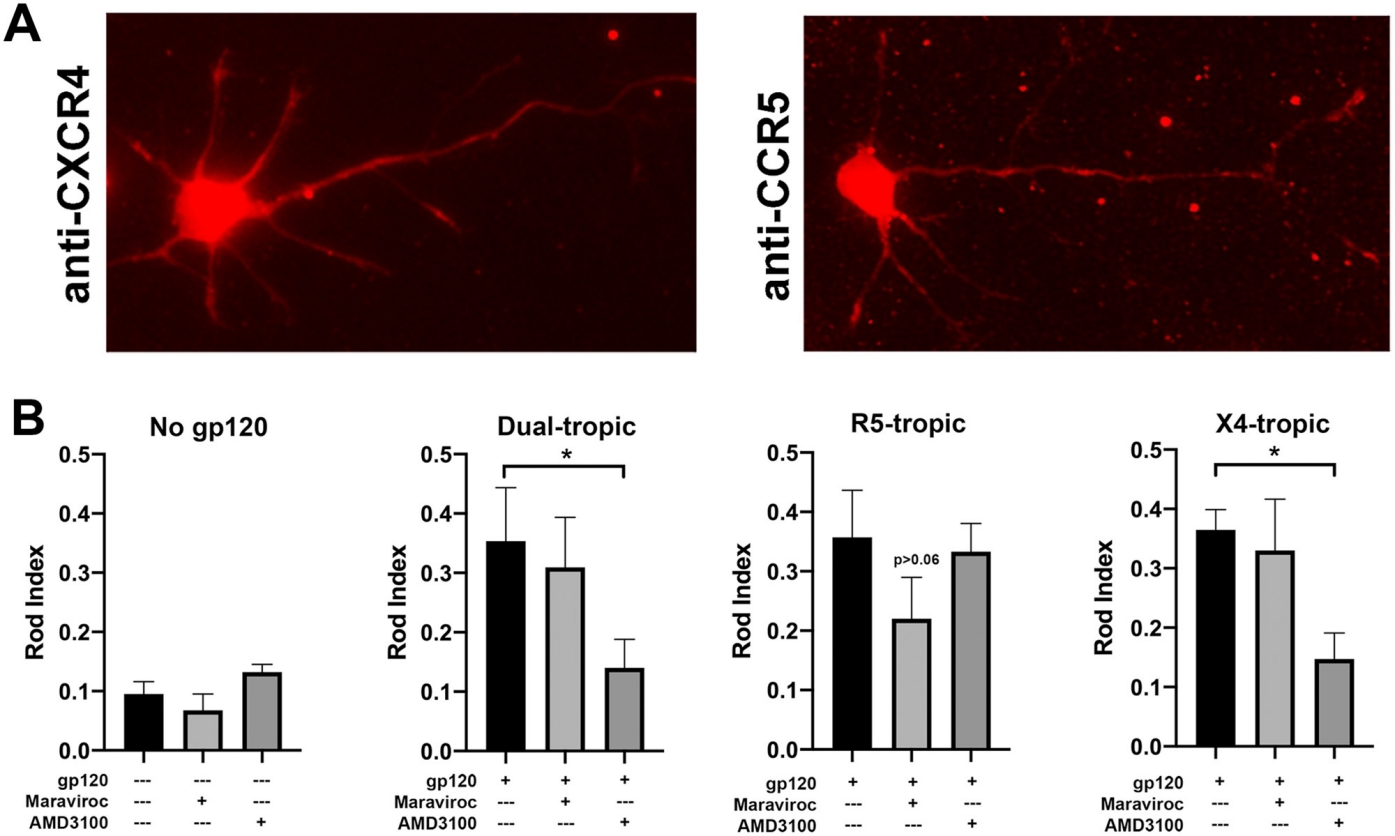
CXCR4 receptors predominantly mediate gp120-dependent rod formation. (A) Mouse hippocampal neurons (C57BI/6) at DIV 7 express both CXCR4 and CCR5 receptors on the surface of neuronal soma and processes as revealed by immunostaining with receptor-specific antibodies in the absence of a permeabilization step. (B) Neuronal cultures were incubated with the CCR5-specific inhibitor maraviroc (100 nM) or the CXCR4-specific inhibitor AMD3100 (50 nM) for 1h prior to and during exposure with 250 pM of either dual- or monotropic gp120. The presence of AMD3100 blocked rod induction by X4-tropic gp120_IIIB_ and dual tropic gp120_MN_ but not by R5-tropic gp120_CM_. In contrast, the CCR5 antagonist maraviroc reduced rod formation in neurons exposed to R5-tropic gp120_CM_ but was ineffective in blocking rod formation in response to X4-tropic gp120_IIIB_ or dual tropic gp120_MN_ (*p<0.01 unless indicated otherwise). Hippocampal neurons obtained from PrP^C^-null mouse line expressed both CXCR4 and CCR5 chemokine receptors indistinguishable from neurons derived from wild type mice (S3 Fig).

Since both dual- and mono-tropic gp120 elicited rod induction, we examined the role of the individual chemokine co-receptors CCR5 and CXCR4. Though most cell types in the human CNS express either chemokine receptor, there has been less consistent evidence of CCR5 expression on neuronal cells [2, 36–38]. First, we confirmed that both CXCR4 and CCR5 are indeed expressed on the surface (soma and all neurites, respectively) of both mouse and rat hippocampal neurons (DIV 7) by immunostaining omitting permeabilization in the staining protocol (Fig 4A). As levels of CCR5 expression in mouse neurons have been reported to increase following exposure extrinsic stress insults [39], we tested whether gp120 exposure may also upregulate CCR5 membrane expression. Mouse hippocampal neurons were treated with 250 pM of R5 and X4-tropic gp120 for 16 h prior to being fixed and immunostained for CCR5 or CXCR4 but no apparent increase in expression of either receptor was detectable as the images were identical in intensity to those in Fig 4A.

Although binding of gp120 to CCR5 and CXCR4 co-receptors is essential for viral envelope fusion with the host membrane, interactions with other neuronal receptors, including N-methyl-D-aspartate receptors (NMDAR) and nicotinic acetylcholine receptors (nAChR) have been reported [16, 40–42]. Having demonstrated that gp120 co-receptors are present in the membrane of rodent hippocampal neurons, we sought to confirm that rod formation is a direct consequence of gp120 interaction with these specific chemokine receptors. To this end, we exposed mouse hippocampal neurons (DIV 6) to dual tropic gp120_MN_, R5-tropic gp120_CM_, or X4-tropic gp120_IIIB_ (250 pM each) in the presence of CCR5- and CXCR4-specific inhibitors maraviroc (100 nM) and AMD3100 (50 nM), respectively (Fig 2B). For dual-tropic gp120_MN_, the presence of AMD3100 significantly reduced the number of rods whereas maraviroc was ineffective. In neurons exposed to R5-tropic gp120_BaL_, the CXCR4 antagonist AMD3100 had no effect but there was a measurable decrease in rod index in cultures exposed to maraviroc but it did not quite reach our level for significance. In neurons exposed to X4-tropic gp120_IIIB_, rod index was not reduced by maraviroc but was reduced to control levels in the presence of AMD3100 supporting the observation that neuronal rod induction is more sensitive to gp120 signaling through CXCR4. Importantly, neither maraviroc nor AMD3100 significantly affected rod induction in cultures exposed to the opposite receptor-binding gp120 strain and, spontaneous rod formation was not affected by the presence of either inhibitor. These findings strongly suggest that rod induction by gp120 through a CXCR4-mediated signal transduction pathway is more potent since blocking CXCR4 with AMD3100 returned rod formation to basal levels for both dual- and X4-tropic gp120.

### Gp120 mediates rod formation through a cellular prion protein PrP^C^-dependent pathway that requires the NOX activation

**Fig 5:**
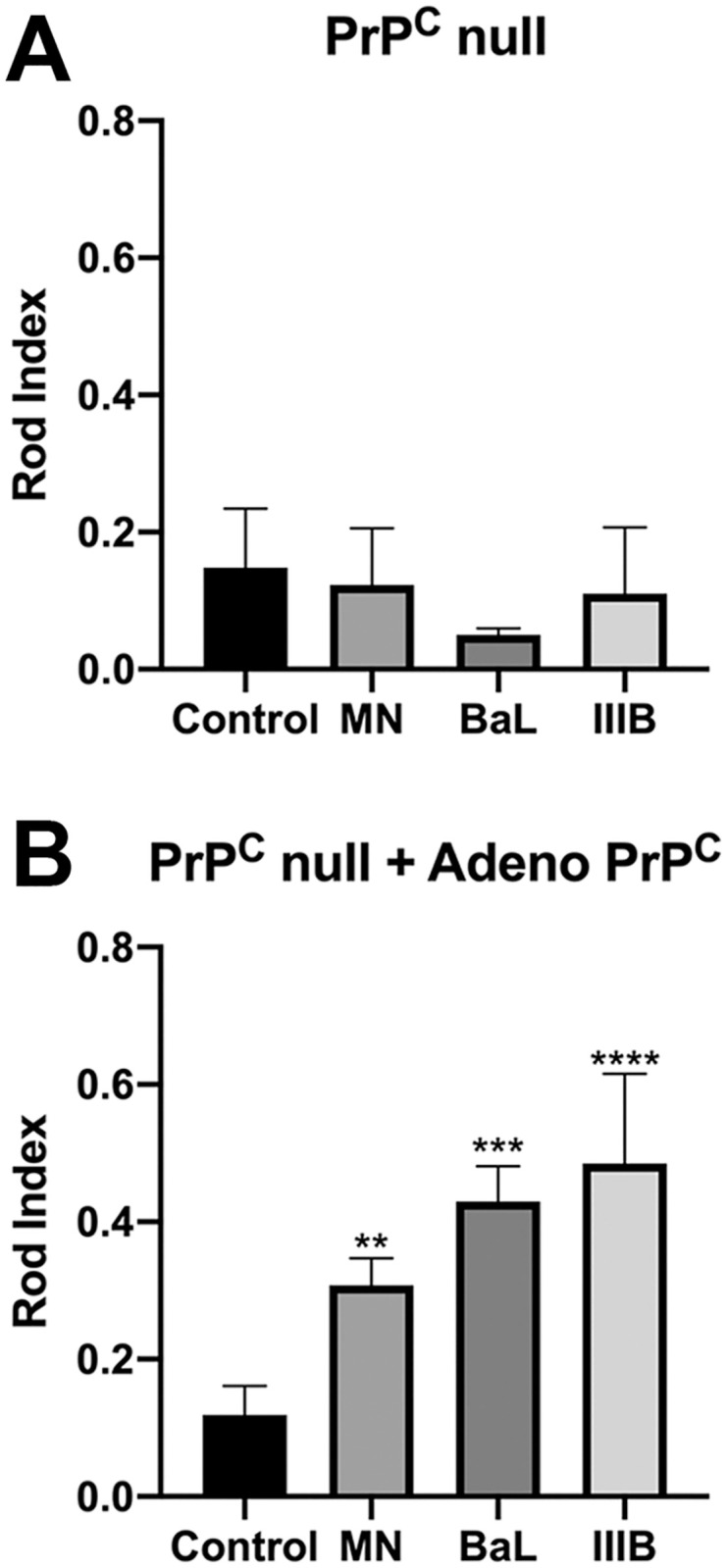
Gp120-mediated rod induction requires the expression of the cellular prion protein PrP^C^. (A) Dissociated hippocampal neurons from PrP^C^-null mice (6 DIV) were expose to dual-tropic gp120_MN_, R5-tropic gp120_BaL_, or X4-tropic gp120_IIIB_ for 16 h (250 pM each) and rod formation was quantified as rod index. The lack of PrP^C^ abolishes rod induction to levels indistinguishable from spontaneous rod formation. (B) PrP^C^-null neurons were infected with adenovirus (50 moi) for expressing EGFP-PrP^C^ or EGFP (control) for 60 h prior to gp120 exposure for an additional 16 h (250 pM, see strains above) followed by rod quantification (rod index) in EGPF-expressing neurons. Restoring PrP^C^ expression resulted in a robust gp120-mediated rod formation above control levels for all tropic forms tested (**p = 0.0053, ***p = 0.0003, ****p<0.0001).

Rod induction by Aβ_d/t_ and proinflammatory cytokines occurs through a PrP^c^-dependent signaling pathway linking these ligands to the activation of NOX [19]. We hypothesized that gp120-mediated rod induction must also require membrane expression of PrP^C^ and active NOX. To test this hypothesis, we exposed hippocampal neurons cultured from PrP^C^ null mice to all three gp120 tropic strains. Unsurprisingly, none of the tested strains of gp120 induced rod formation (Fig 5A). To verify the requirement of PrP^C^ expression for gp120-mediated rod formation, we expressed EGFP-PrP^C^ driven by a CMV promotor in PrP^C^-null hippocampal neurons using recombinant adenovirus, which has previously been demonstrated to drive the expression of functional PrP^C^ at the membrane surface [43]. In neurons re-expressing PrP^C^, 18 h treatment with 250 pM of each tropic strain of gp120 induced a significant increase in rod formation over control (Fig 5B). Having confirmed an essential role for PrP^C^ in gp120-mediated rod formation, we next sought to confirm the requirement for active NOX.

**Fig 6:**
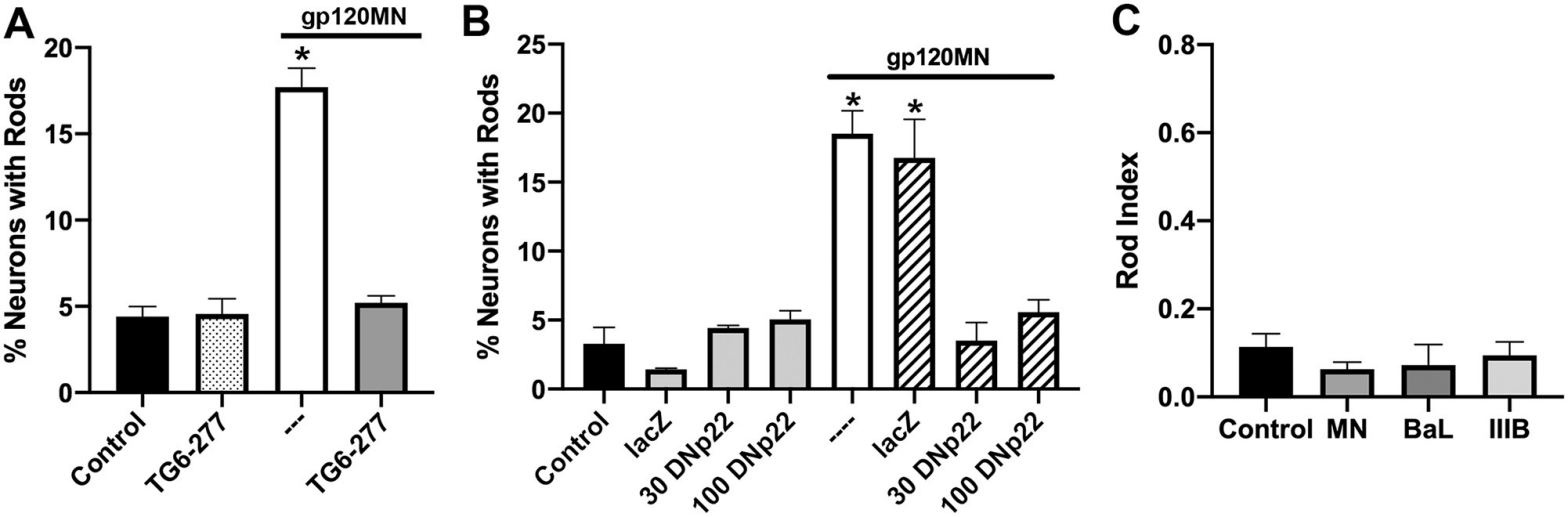
Gp120-induced rod formation requires NADPH oxidase (NOX). NOX activity was inhibited using pharmacological, molecular biological and genetic approaches in dissociated cultures of mouse hippocampal neurons. (A) The NOX inhibitor TG6-277 blocked dual-tropic gp120MN-induced rod formation (16 h exposure, 250 pM) compared to an absence of the pharmacological inhibitor (*p<0.05). TG6-277 alone did not alter basal levels of spontaneous rod formation. (B) Adenoviral-mediated expression of a dominant-negative mutant of the small NOX membrane subunit p22PHOX (DNp22^PHOX^) completely abolished rod formation upon exposure to dual-tropic gp120_MN_ (250 pM) using MOIs (MOI: multiplicity of infection) of 30 to 100. Note, expression of the reporter gene LacZ (control) did not induce rods nor interfere with gp120_MN_-mediated rod formation (*p<0.05). (C) Hippocampal neurons lacking the cytosolic subunit p47^PHOX^ crucial for NOX activity (p47^PHOX^-null mouse line) were exposed to dual-tropic gp120_MN_, R5-tropic gp120_BaL_, or X4-tropic gp120_IIIB_ (250 pM each, 16 h) and rod induction quantified (rod index). Neither gp120 tropic strain induced rod formation above control levels in p47^PHOX^-null neurons.

Active NOX2, a superoxide (O_2_^-^) generating multi-subunit enzyme, is comprised of two membrane subunits (gp91^PHOX^, p22^PHOX^) and three cytosolic components (p47^PHOX^, p67^PHOX^, and p40^PHOX^) in addition to the ancillary small GTPase Rac1. To test the requirement for NOX activity in rod induction by gp120, we employed a combination of pharmacological, molecular, and genetic approaches to block NOX activity (Fig 6). First, we used the NOX inhibitor TG6-227 (kindly provide by Dr. David Lambeth, Emory University GA) and exposed cells to dual-tropic gp120_MN_ (16 h, 250 pM). Rod-induction by gp120_MN_ was significantly reduced in presence TG6-227-treated neurons compared to an absence of the pharmacological inhibitor (Fig 6A). Next, we expressed the dominant-negative mutant of the NOX small membrane subunit p22^PHOX^ (DNp22^PHOX^) in dissociated mouse hippocampal neurons using recombinant adenovirus at 30 and 100 MOI (multiplicity of infection) shown to effectively block NOX activation [44]. DNp22^PHOX^-expressing hippocampal neurons revealed no increase in rod induction when exposed to 500 pM dual-tropic gp120_MN_ at either MOI tested (Fig 6B). In contrast, both control neurons and those infected with adenovirus expressing the lacZ reporter (control infected) responded to dual-tropic gp120_MN_ with a nearly 4-fold increase in rods. Lastly, we demonstrated that the absence of the cytosolic membrane subunit p47^PHOX^ (p47^PHOX^-null mouse line) negated rod formation upon exposure to dual-tropic gp120_MN_, R5-tropic gp120_BaL_, or X4-tropic gp120_IIIB_ (Fig 6C). Hippocampal neurons obtained from p47^PHOX^-null or PrPc-null mouse lines expressed both CXCR4 and CCR5 chemokine receptors indistinguishable from wild type mouse hippocampal neurons (S3 Fig). Together these results demonstrated that the inhibition of NOX activity is sufficient to block gp120-induced rod formation.

### CCR5 and CXCR4 antagonist block rod induction by soluble Aβ_d/t_

**Fig 7:**
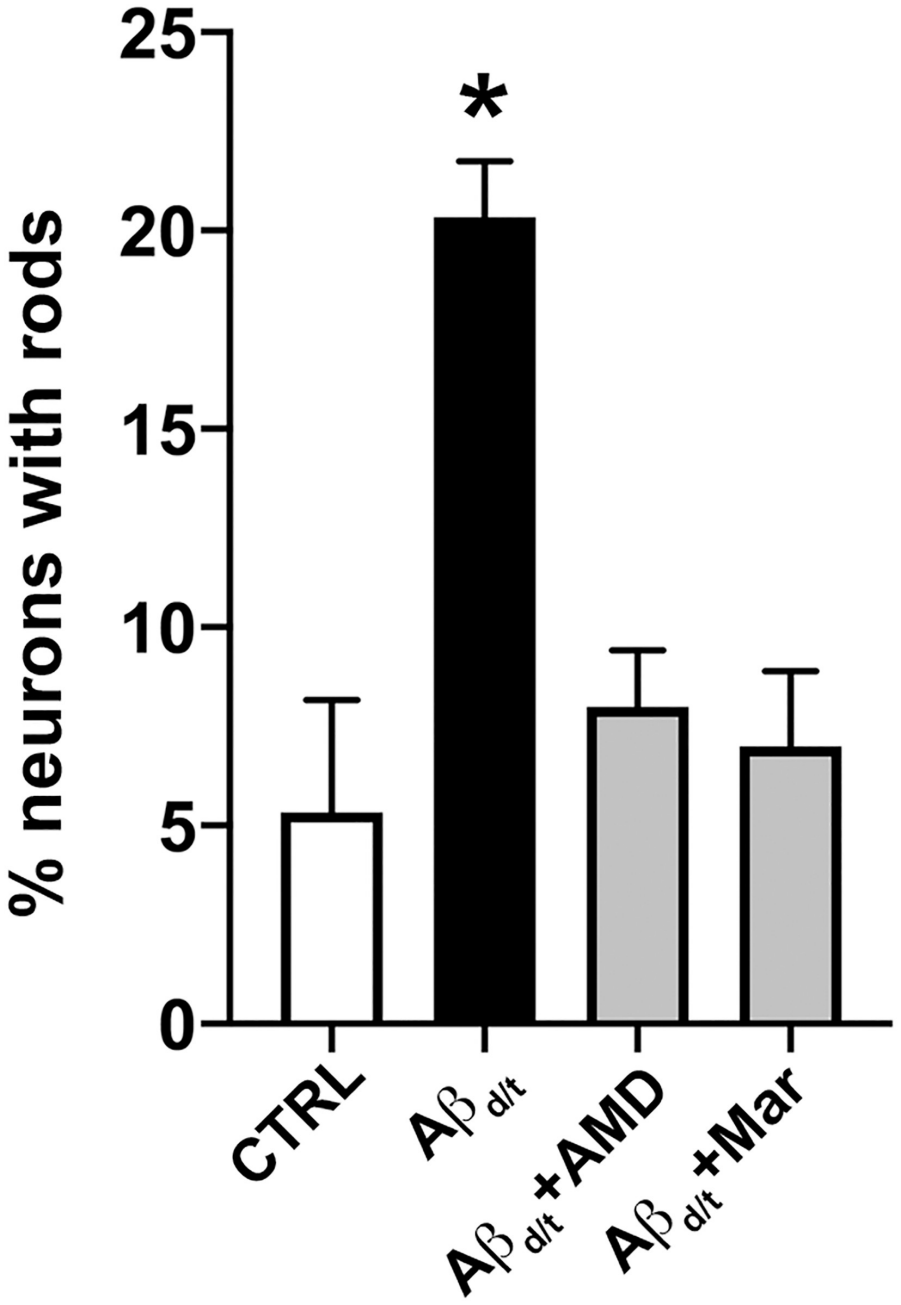
Inhibition of CCR5 and CXCR4 signaling abolishes Aβ_d/t_-mediated rod formation. Dissociated rat hippocampal neurons (DIV 6) were exposed to 1 nM Aβ_d/t_ for 16 h followed by immunostaining against cofilin and rod quantification. As expected, the presence of 1 nM Aβ_d/t_ induced a 4-fold increase in rod-containing neurons compared to control (Con), which was abolished by the presence of either the CXCR4 or CCR5 inhibitors, AMD3100 (60 nM) or maravirac (60 nM), respectively (*p<0.05).

Our previous studies demonstrated the rod-inducing capacity of soluble Aβ_d/t_ [19]. Although several receptors for Aβ_d/t_ in the CNS have been identified including acetylcholine receptor and PrP^C^, recent reports imply a significant role of CCR5 activation in the progression and acceleration in the development of AD [45]. A decline in expression of CXCL12, the natural antagonist of CXCR4, occurs in AD and is linked to neurocognitive events both on the behavioral and molecular level revealing a Rac1-dependent effect on actin polymerization [46, 47]. Therefore, we examined whether Aβ_d/t_-mediated rod induction implicated the CCR5 and/or CXCR4 receptors (Fig 7). Dissociated cultures of rat hippocampal neurons (DIV 6) were exposed to Aβ_d/t_ (1 nM, 16 h) in the presence or absence of the CXCR4 antagonist AMD3100 (100 nM) or the CCR5 antagonist maraviroc (50 nM). Interestingly, inhibition of either chemokine receptor abolished Aβ_d/t_-mediated rod induction. This finding suggests a potential direct interaction of Aβ_d/t_ with CCR5 and CXCR4 receptors or via a promiscuous co-receptor such as PrP^C^.

## Discussion

Cofilin-actin rod induction is a cellular response to many neurodegenerative stimuli including mitochondrial dysfunction, ischemia/reperfusion, NMDA receptor-mediated excitotoxicity, pro-inflammatory cytokines, as well as Aβ_d/t_ [4, 19, 48–50]. Here we describe for the first time HIV gp120-mediated induction of cofilin-actin rods in mouse hippocampal neurons requiring interactions with CCR5 or CXCR4 chemokine receptors linked to a pathway necessitating both PrP^C^ and NOX. This novel mechanism potentially contributes to neuronal dysfunction in HAND.

Common to all rod inducers is the activation of NOX, which produces ROS, causal to rod formation. Gp120-mediated rod formation was abolished by pharmacological inhibition, the introduction of dominant-negative mutation in the p22^PHOX^ membrane subunit, or by a gene-knockout of cytosolic subunit p47^PHOX^ [51]. Although p22^PHOX^ does not contain catalytic function, the dominant negative mutant interferes with NOX2/p47^PHOX^ interactions [44]. The three cytosolic subunits p67^PHOX^, p40^PHOX^, and p47^PHOX^ always exist as a complex and a lack of p47^PHOX^ disables complex formation and its key role in NOX activation [52, 53]. Similarly, PrP^C^ was essential for rod generation in response to gp120 exposure. Re-expression of PrP^C^ successfully recovered the ability of gp120 to induce rods in PrP^C^-null mouse hippocampal neurons. Importantly, we found that cofilin-actin rods induced by Aβ_d/t_ also occurs via a maraviroc/AMD3100-sensitive pathway implicating a contribution of CCR5 and CXCR4 chemokine receptors in AD, which we previously showed involves a PrP^C^/NOX-dependent signaling pathway [19]. These results provide evidence that rod induction via the PrP^C^/NOX-dependent pathway downstream of CCR5 and CXCR4 receptors may be a common mechanism underlying neuronal impairment observed for several neurodegenerative diseases, including Alzheimer’s disease and HAND.

**Fig 8:**
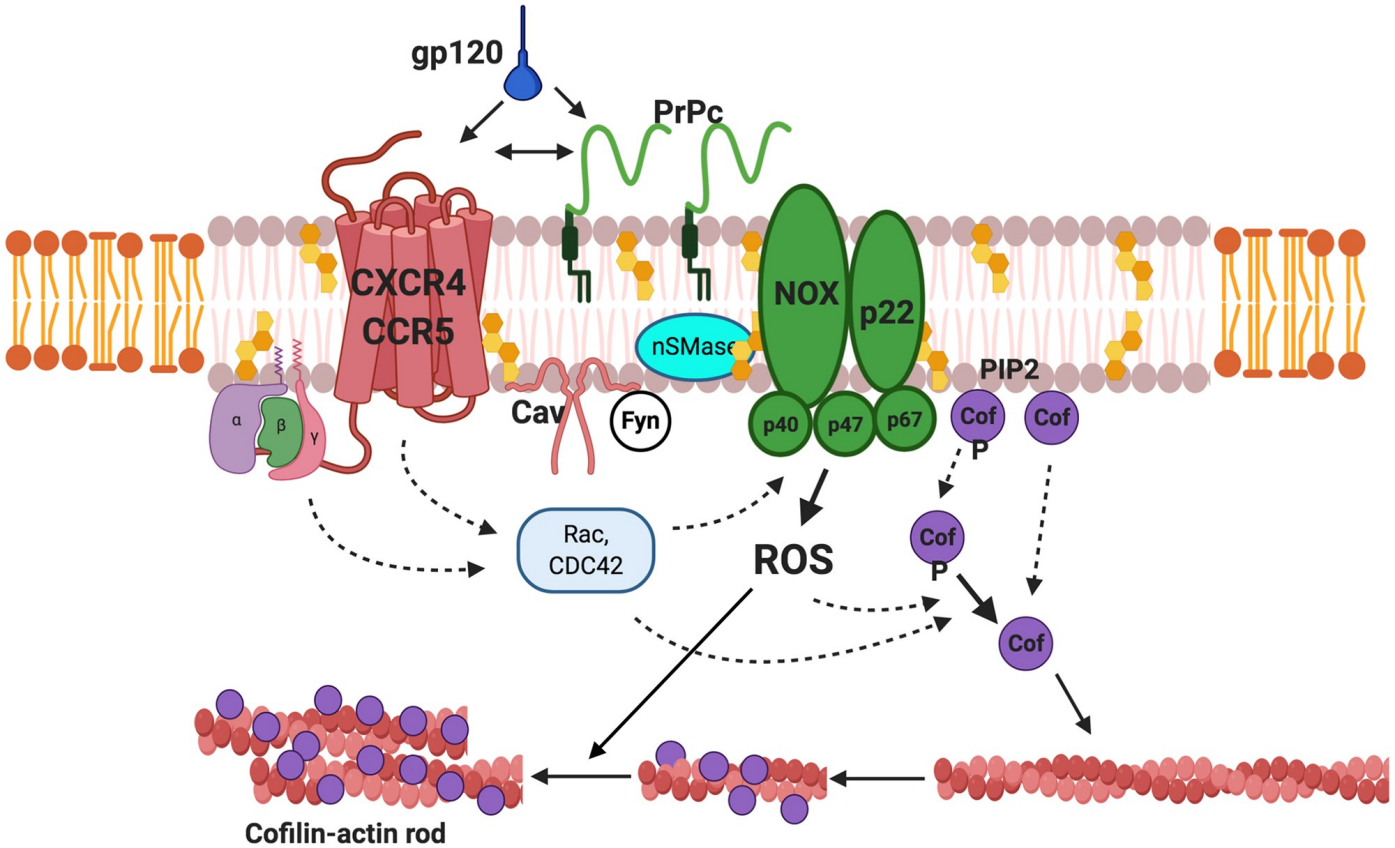
Signaling components in gp120-mediated cofilin-actin rod formation and some putative downstream effectors. PrP^C^ is associated with lipid-rafts, plasma membrane domains enriched in cholesterol, gangliosides, sphingolipids and phosphatidyl inositol phosphates (PIP2) all of which properly organize chemokine receptors (CXCR4, CCR5), large and small membrane subunits of NADPH oxidase (NOX and p22^PHOX^, respectively), and other raft proteins such as caveolin (Cav), neutral sphingomyelinase (nSMase), and fyn, a src-family kinase. Interaction of gp120 with chemokine receptors and potentially PrP^C^ stimulates NOX activity to generate ROS through one or more signaling pathways likely involving heterotrimeric G-proteins, Rho family GTPases, caveolin, and fyn. The small GTPase Rac1 is essential for NOX activation whereas Cdc42 is implicated in cofilin activation [66]. The oxidative environment favors activation of the cofilin phosphatase slingshot-1L [67] and dephosphorylation of inactive, phospho-cofilin (pCof) to the active form (Cof). Alterations in phosphoinositides can release PIP2-sequestered phospho and dephospho-cofilin [68]. The increasing pool of active cofilin can locally saturate F-actin, sever filaments, and form cofilin-saturated F-actin fragments now susceptible to ROS-induced formation of intermolecular disulfide-linked cofilin dimers ultimately forming thick bundles of cofilin-actin filaments or rods [20]. Note, lipid rafts (grey lipid bilayer) are thicker in diameter compared to the more fluid plasma membrane (orange bilayer). Dashed lines indicate multi-step pathways whereas solid lines refer to single-step pathways.

Although PrP^C^ and NOX are critical for rod induction, the signal transduction pathway by inference is likely much more complex (Fig 8). The importance of the membrane lipid raft domain as a regulator of protein structure and function cannot be overstated and is key to the gp120-mediated pathway to rod formation [13, 54]. For instance, cholesterol modulates protein function through interactions with a cholesterol recognition amino acid consensus sequence as a ‘chaperone-like’ allosteric regulator [55, 56]. Notably, putative cholesterol binding sites have been identified in the structure of both CCR5 and CXCR4, supporting the necessity of lipid raft localization for proper receptor function [57, 58]. Furthermore, both PrP^C^ and NOX2 are localized to raft domains where raft composition similarly affects conformational stability and enzyme activity [59–61]. In a passive role, PrP^C^ organizes lipid raft domains rich in cholesterol, sphingolipids, and cytoplasmic phosphatidylinositol phosphates crucially impacting CCR5 and CXCR4 receptor activity and possibly NOX activity as well. More directly, PrP^C^ is known to insert a domain of its octapeptide repeat region into the lipid raft membrane to interact with the cytoplasmic leaflet-associated caveolin, which can modulate src-family kinase signaling [62, 63] such as Fyn, which phosphorylates p47^PHOX^ implicated in NOX activation [64]. Furthermore, PrP^C^ is a well-documented promiscuous co-receptor and could serve this function in gp120-chemokine receptor interactions. Jana et al. (2004) [13] described the activation of neutral sphingomyelinase (nSMase) by gp120 in neurons increasing ceramide and ceramide-1-phosphate levels, which are both lipid activators for NOX [65].

The importance of the host cytoskeleton to the HIV life cycle imparts a key role for actin in viral entry, replication and assembly, and budding [69–71]. Modulation of actin regulatory proteins in host cells is one strategy [72]. Activation of pathways linked to cofilin have been described in resting CD4+T cells facilitating viral post-entry nuclear import critical in establishing HIV latency [73]. Notably, HIV does not productively infect neurons yet individual HIV proteins affect neuronal cytoskeleton dynamics through modulating the activity of regulatory proteins including cofilin but the outcomes likely differ from those associated with productive infection [74]. Formation of cofilin-actin rods rest on two prerequisites: 1) the presence of active, dephosphorylated cofilin, and 2) an oxidative environment. In HIV patients, downregulation of cofilin phosphorylation has been reported [75, 76]. Although excess dephosphorylated cofilin could account for rod formation [77], a vast pool of dephosphorylated cofilin is present in adult mouse brain accounting for up to 95% of total cofilin with no apparent formation of rods (S4 Fig). Presumably, most active, dephosphorylated cofilin is sequestered such as that bound to phosphatidylinositol phosphates enriched in lipid raft domains, or bound to F-actin regulating myosin II and thus contractile events, which run amuck in cells where ADF/cofilin expression has been silenced [78]. Thus, dephosphorylation of a phospho-cofilin pool is not inherently necessary for rod formation. Rod formation appears to be intimately linked to an oxidative environment that favors release of sequestered cofilin. Isolation of cofilin from rods formed by ATP-depletion predominantly exists as disulfide-linked dimers [20], but other oxidation products of cofilin have not been further investigated. Moreover, ROS produced by NOX can simultaneously activate slingshot-1, a phospho-cofilin phosphatase, by oxidizing and removing an inhibitory 14-3-3 family protein that helps maintain slingshot-1 in an inactive state [67]. Cofilin’s collaboration with Aip1/WDR1 supports actin filament severing and recruitment of oligomers of CAP1/2 to cofilin-actin fragments assists in their complete depolymerization [79, 80].

Neuronal injury observed in HAND occurs indirectly through the release of neurotoxic, proinflammatory host factors predominantly from microglia and astrocytes, which are productively infected by HIV [2, 31, 32], or as a direct neurotoxic effect of soluble HIV proteins shed from infected host cells and virus such as gp120 [12, 33, 34, 81]. Pertinent to our studies, gp120 stimulates the release of pro-inflammatory and pro-apoptotic cytokines such as TNFα [82–84]. Although our cultures of dissociated mouse hippocampal cultures were virtually devoid of microglia cells (see Fig 3), a significant population of astrocytes is present (approximately 40% of cells). In the case of Aβ_d/t_ as the stressor, secretion of rod-inducing levels of proinflammatory cytokines was not detectable [19]. In pure astrocyte cultures or mixed astrocyte/microglia cultures in serum-containing conditions, gp120-dependent secretion of TNFα, Il-1β, Il-6, and Il-8 ranged from 200 to 600 pg/ml [85–87]. Significant rod induction in dissociated hippocampal neuron cultures requires a minimum cytokine concentration of 5 ng/ml nearly 10 times what is produced by pure astrocyte cultures. Thus it is unlikely that gp120-induced astrocyte-released cytokines could make a significant contribution to rod formation. Considering these arguments and findings, a direct mechanism in which gp120 interacts with CCR5 and/or CXCR4 receptors on the neuronal membrane is most likely to accounts for the PrP^C^/NOX-mediated pathway of rod induction.

Although signaling through either CCR5 or CXCR4 induced a significant increase in rod index, there appear to be differences in the strength of induction associated with individual receptors. Whereas AMD3100 inhibition of the CXCR4 receptor significantly reduced rod induction to control levels for both dual-tropic and X4-tropic strains of gp120, maraviroc at 100 nM achieved a partial yet non-significant reduction in rod formation by dual-tropic or R5-tropic gp120. This concentration commonly used in the literature is not associated with cytotoxicity for the times of our exposure. However, 20 μM maraviroc accomplished significant rod reduction yet widespread neurotoxicity was observed allowing the possibility that 100 nM maraviroc is insufficient to block all CCR5 receptor signaling. Our data conclusively demonstrates that different tropic-strains of gp120 are indeed receptor specific. Immunocytochemistry demonstrated the presence of CCR5 and CXCR4 in the plasma membrane of dissociated mouse hippocampal neurons (Fig 4 and S3 Fig) in accordance to findings on rat cortical neurons [88]. However, expression levels for different antigens cannot be inferred from immunoreactivity due to varying antibody affinities. A more quantitative study by Petito et al. (2001) found similar levels of CXCR4 and CCR5 on human hippocampal neurons (postmortem control) whereas hippocampal neurons from AIDS individuals revealed increased CXCR4 expression accompanied by decreased CCR5 [89]. Interestingly, CCR5 expression was absent in CA1 hippocampal regions. Similar mRNA expression levels for CCR5 and CXCR4 were reported in human embryonic neurons and rat hippocampal neurons [90, 91]. Most studies provide relative mRNA levels for each chemokine receptor and thus do not allow a comparison of the protein expression [92].

Synaptopathy has emerged as a hallmark of HIV-mediated neurotoxicity of HAND in the post-cART era [74] and impairments in neuronal cytoskeleton are highly relevant driven by neurotoxic HIV proteins [93–95]. Synaptic dysfunction and dendritic simplification are linked to gp120 neurotoxicity and underlie cognitive impairments observed in HAND yet the mechanisms are poorly understood [12, 96, 97]. The activation of a PrP^C^/NOX-mediated pathway of rod induction is one potential mechanism for gp120-induced synaptic dysfunction involving the perturbation of neuronal cytoskeleton dynamics. Although rod pathology has not been described in postmortem brain of HAND patients, perhaps because it requires non-standard procedures for their immunostaining [17, 22], persistent cofilin-actin rods have been observed during the progression of Alzheimer’s [4, 22, 98] and following ischemia [48, 49], correlating strongly with cognitive impairment [99, 100]. Common to these cofilin-actin pathologies [101, 102], synaptic dysfunction might arise from either or both an interruption of vesicular transport due to occluded neurites [21, 77] or a sequestration of cofilin from dendritic spines blunting its role in post-synaptic plasticity [23, 103, 104]. Only recently, dendritic simplification and cognitive flexibility in a transgenic rat model of HIV-1 infection was rescued by CXCL12, an endogenous chemokine and antagonistic ligand for the CXCR4 receptor, presumably outcompeting gp120 interaction with CXCR4 [46]. Marchionni et al. (2012) reported opposite outcomes on spontaneous activity of Cajal-Retzius cells from CXCL12 and gp120 treatment [105]. A significant role for CCR5 was implied in Aβ_d/t_-dependent synaptotoxicity responsible for neurocognitive deficits in AD [45]. Administration of the CXCR4 antagonist AMD3100 to 3xTg-AD mice improve AD pathologies, neuroinflammation, and cognition [63]. Lastly, directly increasing cofilin phosphorylation in cultured neurons decreased rod formation in response to Aβd/t and in mouse models of AD and other neurodegenerative diseases lead to marked cognitive and behavioral improvements [106].

Taken together, inhibition of cofilin-actin rod formation by the CXCR4 and CCR5 antagonists AMD3100 and maraviroc along with the promiscuous nature of PrP^C^ and NOX activity and their essential role in signaling downstream of both Aβd/t and gp120, strongly suggests that rod formation is of central importance to synaptopathy and cognitive decline observed in HAND and AD. Whether cognitive deficits are a result of direct effects impeding intraneurite vesicular transport [21, 77], sequestering of cofilin to inhibit its role in dendritic spine dynamics [23, 104], or other actin-based functions required for normal functions of neuronal networks remains to be determined. Therapeutics, including chemokine receptor antagonists that target rod formation, could ameliorate not only HAND but also pathologic rod formation in response to Aβ oligomers and proinflammatory cytokines. Since lipid rafts have also been implicated in the pathology of several neurodegenerative disorders including HAND, modulation of membrane architecture could be employed as another target to blunt signaling events associated with rod formation.

## Supporting information

S1 Fig Effects of L-cysteine concentration of spontaneous and induced rod formation in dissociated hippocampal neurons. (A) Spontaneously formed rods were quantified per field of view in DIV 6 cultures of dissociated mouse hippocampal neurons grown in commercial neurobasal (NB) containing 260 μM L-cysteine (L-cys) as opposed to selfmade NB with concentrations of L-cys of 50 μM, 100 μM, or 260 μM. (B) Percent of neurons with rods induced by neurodegenerative signals or glutamate compared to control (CTRL, spontaneous rods) as a function of L-cysteine concentration. Averages of duplicate samples with range shown by bar. Overnight treatments: Aβ_d/t_ at 1 μM, gp120_MN_ at 500 pM, TNFα at 50 ng/ml. Glutamate at 200 μM was used for 30–60 min.
(TIF)

S2 Fig Turnover of cofilin on filamentous actin bundles in growth cones and rods in neurites. (A) Images of fluorescence recovery after photobleaching (FRAP) for R21Qcofilin-mRFP (arrows) along actin bundles in growth cones (top row) and rods in neurites (bottom row) in DIV 6 hippocampal neurons treated for 24 h with 1 nM Aβ_d/t_. Laser intensity and duration was set to achieve about 80% bleach. Note, R21Qcofilin-mRFP recovery to 50% on actin bundles in growth cones occurs within one minute. In contrast, R21Qcofilin-mRFP recovery to 50% on rod actin bundles is over one hour. (B) FRAP recovery times to 50% of cofilin-RFP on actin bundles starting value from five independent observations each of rods in neurites and in growth cones (GC). Bar for rod recovery represents the range of times determined from extrapolation of curves over a 20 min observation period.
(TIF)

S3 Fig Hippocampal neurons from PrP^C^- and p47^PHOX^-null mice express CXCR4 and CCR5 chemokine receptors. Dissociated cultures of hippocampal neurons derived from (A) PrP^C^- and (B) p47^PHOX^-null mice lines were cultured for 7 days prior to fixation. Omitting permeabilization, cultures were immunostained for either CXCR4 or CCR5 chemokine receptors. Hippocampal neurons expressed both chemokine receptors on neuronal cell bodies and processes. Chemokine receptor expression was indistinguishable from that of wild type neurons considering the application of identical antibody dilutions and image acquisition parameters.
(TIF)

S4 Fig The predominant form of cofilin in brain is active, dephospho-cofilin. Extracts of brain cortex were prepared from six individual adult mice in the presence of phosphatase inhibitors and SDS as described previously and immediately heated in a boiling water bath [25]. Proteins were precipitated with methanol/chloroform [107], and solubilized in 9.5 M urea, 18 mM dithiothreitol, and 2% IGEPAL CA-630 for protein assay [108]. To insure linearity of quantification from blots, loading of 10, 20, 30 and 40 μg of protein were performed. Shown here are the blots from 20 μg protein loads on IPGphor pH3-10 strips (Amesham), transferred after focusing 3 hr to 15% isocratic polyacrylamide gels. Following SDS-PAGE, proteins were transferred to nitrocellulose. After blocking, cofilin and ADF were visualized with a pan rabbit antibody that is equally reactive to both mammalian cofilin-1 and ADF [25]. Positions of ADF and cofilin species were previously identified [109] using antibody to cofilin [110] and an ADF/cofilin phosphospecific antibody [25]. In embryonic chick brain (E9-E19), phosphorylated forms of ADF and cofilin accounted for about 25% of the total ADF/cofilin pool [111].
(TIF)
