# Supplementary figures and images for: Direct interaction of HIV gp120 with neuronal CXCR4 and CCR5 receptors induces cofilin-actin rod pathology via a cellular prion protein- and NOX-dependent mechanism
Source: PLoS One. 2021 Mar 11;16(3):e0248309. doi: 10.1371/journal.pone.0248309 (PMC7951892; doi:10.1371/journal.pone.0248309)

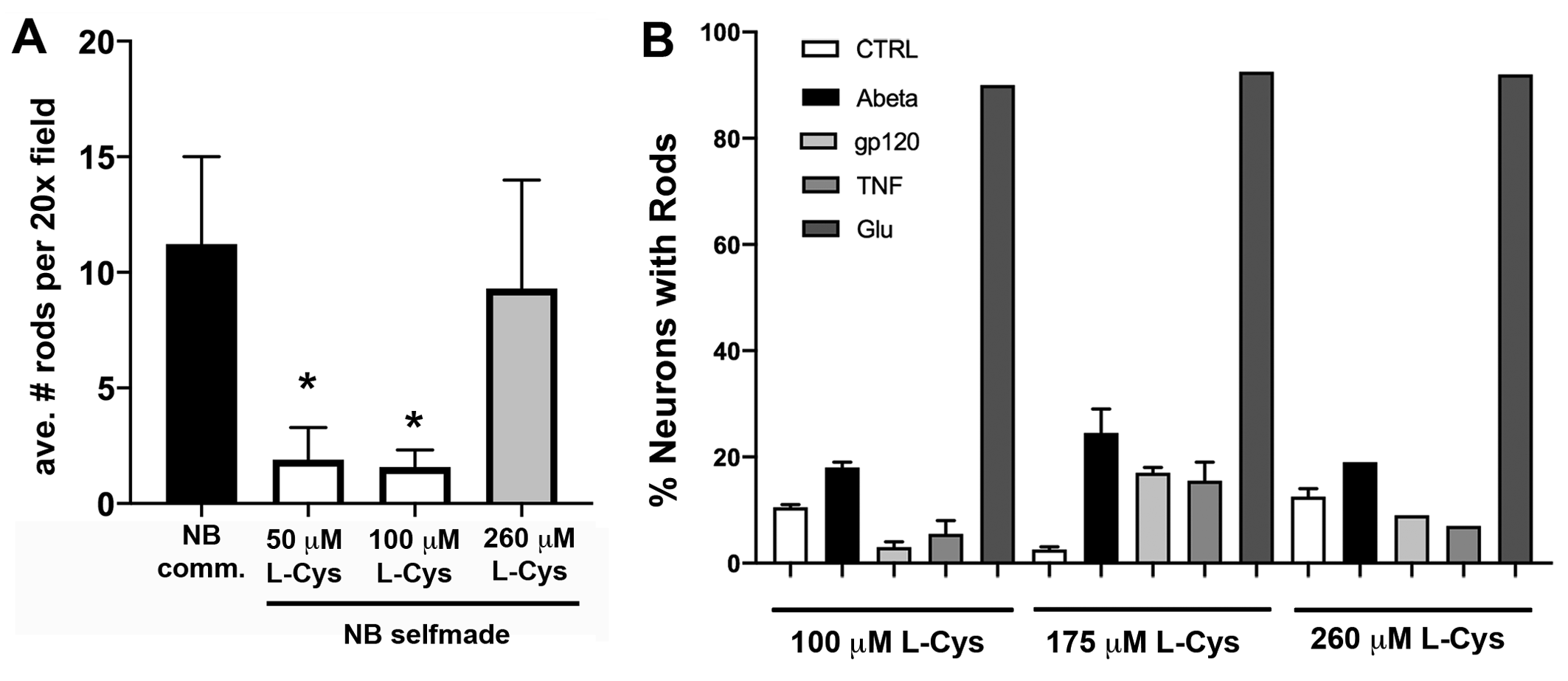

Supplement: S1 Fig — (A) Spontaneously formed rods were quantified per field of view in DIV 6 cultures of dissociated mouse hippocampal neurons grown in commercial neurobasal (NB) containing 260 μM L-cysteine (L-cys) as opposed to selfmade NB with concentrations of L-cys of 50 μM, 100 μM, or 260 μM. (B) Percent of neurons with rods induced by neurodegenerative signals or glutamate compared to control (CTRL, spontaneous rods) as a function of L-cysteine concentration. Averages of duplicate samples with range shown by bar. Overnight treatments: Aβd/t at 1 μM, gp120MN at 500 pM, TNFα at 50 ng/ml. Glutamate at 200 μM was used for 30–60 min. (TIF) [file pone.0248309.s001.tif]

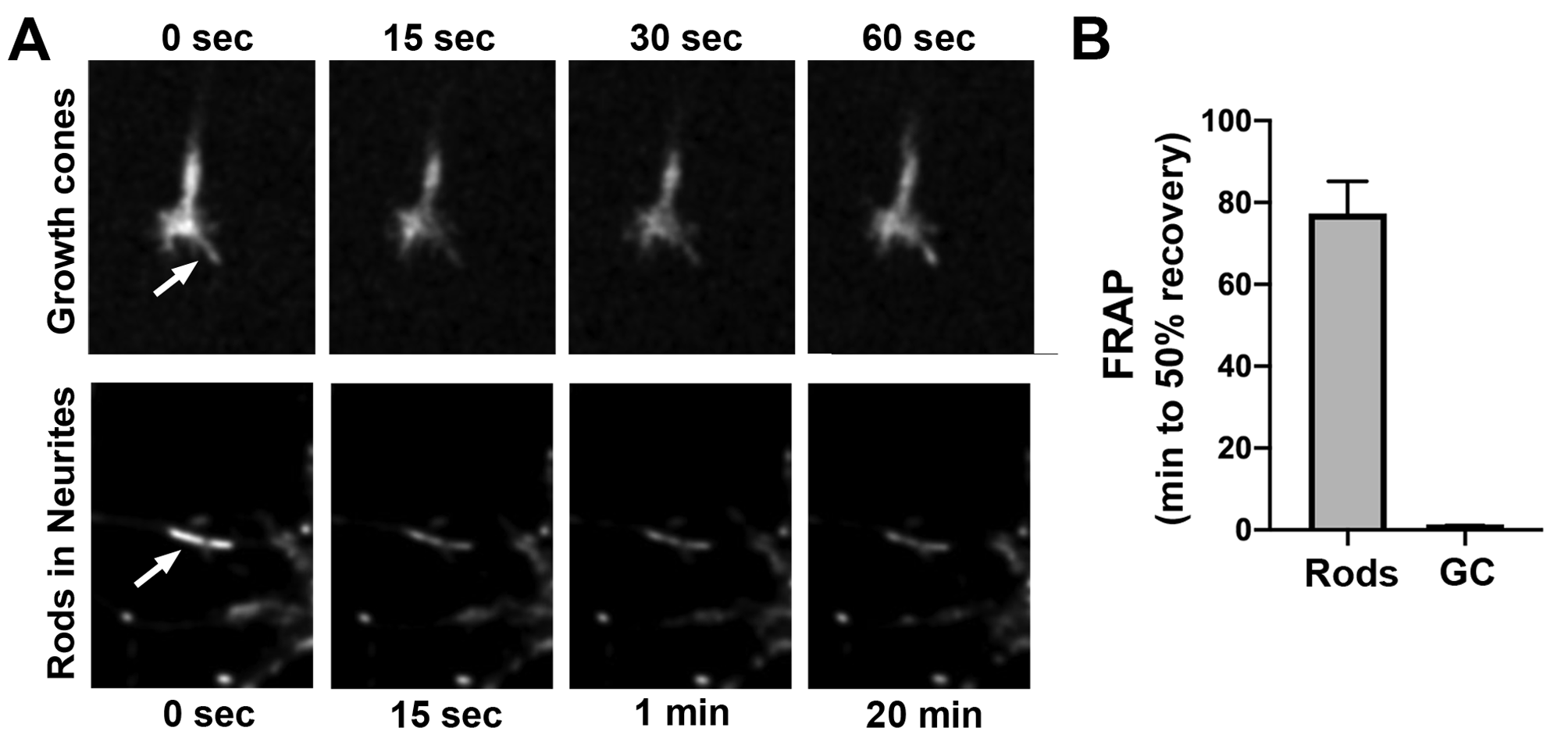

Supplement: S2 Fig — (A) Images of fluorescence recovery after photobleaching (FRAP) for R21Qcofilin-mRFP (arrows) along actin bundles in growth cones (top row) and rods in neurites (bottom row) in DIV 6 hippocampal neurons treated for 24 h with 1 nM Aβd/t. Laser intensity and duration was set to achieve about 80% bleach. Note, R21Qcofilin-mRFP recovery to 50% on actin bundles in growth cones occurs within one minute. In contrast, R21Qcofilin-mRFP recovery to 50% on rod actin bundles is over one hour. (B) FRAP recovery times to 50% of cofilin-RFP on actin bundles starting value from five independent observations each of rods in neurites and in growth cones (GC). Bar for rod recovery represents the range of times determined from extrapolation of curves over a 20 min observation period. (TIF) [file pone.0248309.s002.tif]

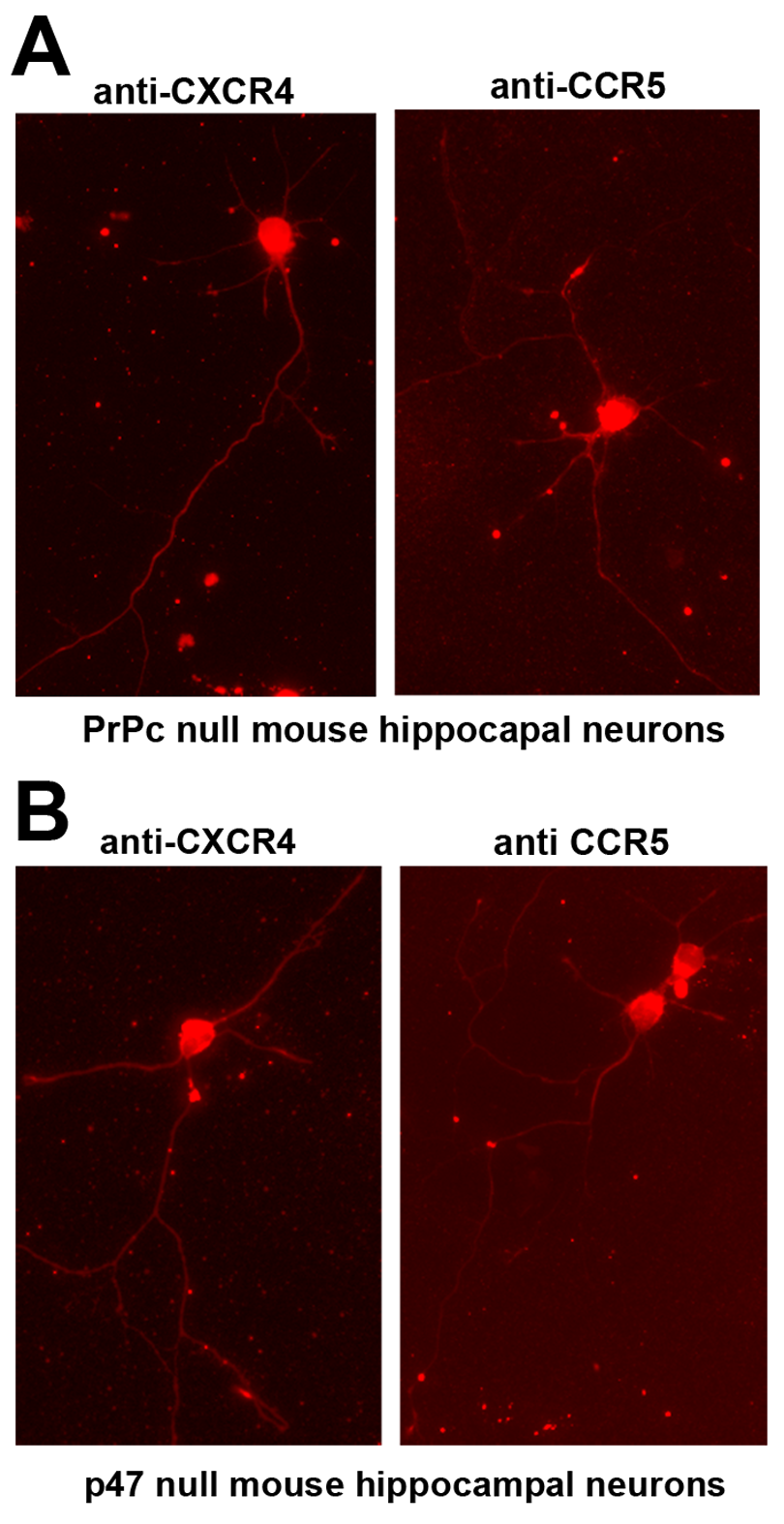

Supplement: S3 Fig — Dissociated cultures of hippocampal neurons derived from (A) PrPC- and (B) p47PHOX-null mice lines were cultured for 7 days prior to fixation. Omitting permeabilization, cultures were immunostained for either CXCR4 or CCR5 chemokine receptors. Hippocampal neurons expressed both chemokine receptors on neuronal cell bodies and processes. Chemokine receptor expression was indistinguishable from that of wild type neurons considering the application of identical antibody dilutions and image acquisition parameters. (TIF) [file pone.0248309.s003.tif]

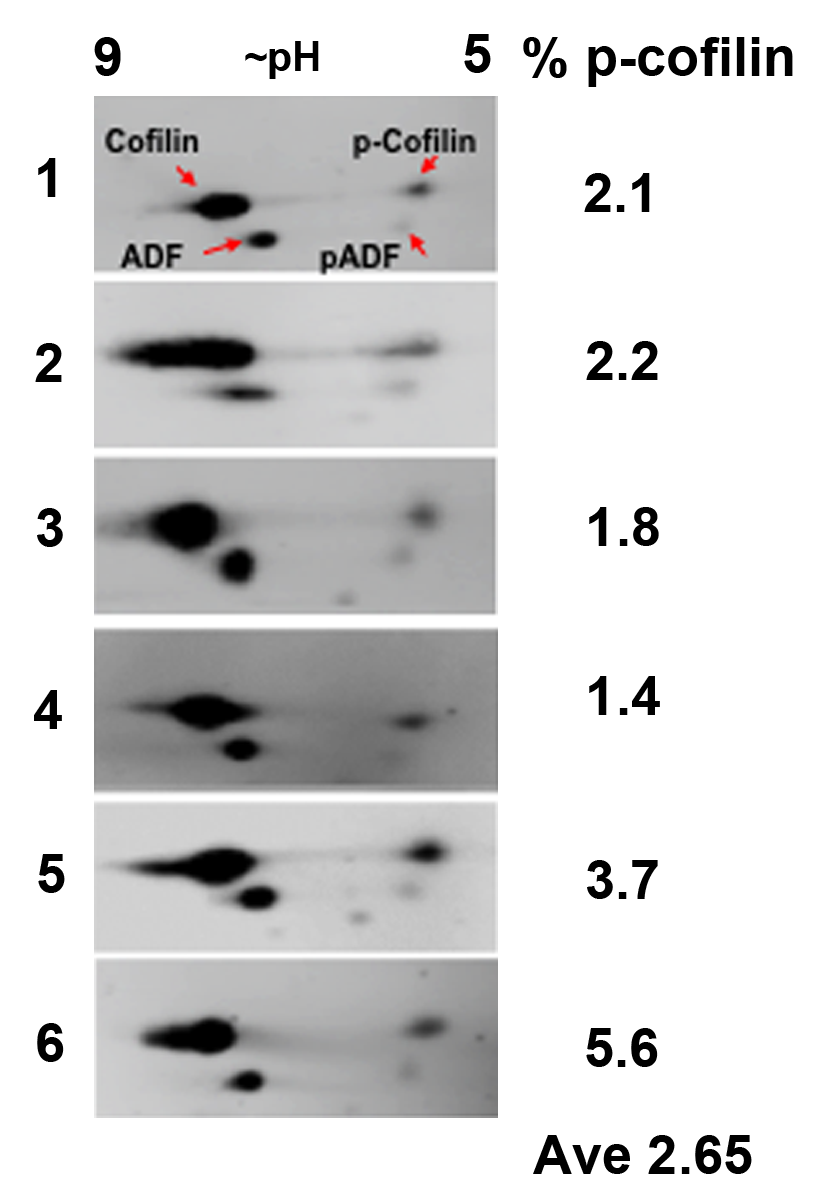

Supplement: S4 Fig — Extracts of brain cortex were prepared from six individual adult mice in the presence of phosphatase inhibitors and SDS as described previously and immediately heated in a boiling water bath [25]. Proteins were precipitated with methanol/chloroform [107], and solubilized in 9.5 M urea, 18 mM dithiothreitol, and 2% IGEPAL CA-630 for protein assay [108]. To insure linearity of quantification from blots, loading of 10, 20, 30 and 40 μg of protein were performed. Shown here are the blots from 20 μg protein loads on IPGphor pH3-10 strips (Amesham), transferred after focusing 3 hr to 15% isocratic polyacrylamide gels. Following SDS-PAGE, proteins were transferred to nitrocellulose. After blocking, cofilin and ADF were visualized with a pan rabbit antibody that is equally reactive to both mammalian cofilin-1 and ADF [25]. Positions of ADF and cofilin species were previously identified [109] using antibody to cofilin [110] and an ADF/cofilin phosphospecific antibody [25]. In embryonic chick brain (E9-E19), phosphorylated forms of ADF and cofilin accounted for about 25% of the total ADF/cofilin pool [111]. (TIF) [file pone.0248309.s004.tif]
